# Anatomy-Based Framework for Anterior and Posterior Cerebral Artery Infarction Patterns in Subfalcine and Uncal Herniation

**DOI:** 10.21315/mjms-04-2026-244

**Published:** 2026-06-30

**Authors:** Puong You Law, Saktivadivelu Letchmanan, Azwa Maulat Kamarul Bahri, Nishanthi Apparow, Suzuanhafizan Omar, Jafri Malin Abdullah

**Affiliations:** 1 Department of Neurosciences and Brain and Behaviour Cluster, School of Medical Sciences and Hospital Pakar USM, Universiti Sains Malaysia Health Campus, 16150 Kubang Kerian, Kota Bharu, Kelantan, Malaysia; 2 Department of Neurosurgery, Hospital Sultanah Nur Zahirah, Jalan Sultan Mahmud, 20400 Kuala Terengganu, Terengganu, Malaysia

**Keywords:** subfalcine, uncal, herniation, anterior cerebral artery, posterior cerebral artery

## Abstract

Raised intracranial pressure within the rigid cranial vault can lead to brain herniation and may be associated with anatomically patterned territorial infarction. However, the relationship between staged brain displacement and regional arterial compromise remains incompletely integrated with the underlying neuroanatomy. We therefore propose an anatomy-based framework centred on the anterior and posterior cerebral artery that links the arterial morphology, regional anatomical variations and stage-dependent displacement in subfalcine and uncal herniation. This framework may help clinicians anticipate herniation-related ischaemic patterns on imaging and provides a practical model for computed tomography interpretation and early recognition of vascular compromise.

## Introduction

Brain herniation results in rapid clinical deterioration and is associated with high mortality rates ranging from 30% to over 70% in the advanced stages (1, 2). Among the supratentorial herniation patterns, subfalcine and uncal herniations are the most prevalent, accounting for ~ 40% to 60% and 25% to 35% of cases, respectively (3, 4). Conventional descriptions of herniation syndromes primarily emphasise gross anatomical displacement and clinical manifestations (2–4) but less often relate the direction of tissue shift to specific arterial territories at risk. In this article, we propose an anatomy-based framework to anticipate how staged subfalcine and uncal herniation may relate to anterior cerebral artery (ACA) and posterior cerebral artery (PCA) compromise.

Clinically, this framework may assist with computed tomography (CT) interpretation in patients with raised intracranial pressure (ICP) by highlighting which arterial territories are vulnerable at different stages of displacement. It may also help clinicians recognise early herniation-related ischaemia and integrate radiological signs with the urgency of clinical decision-making.

## Core Neuroanatomy

Infarction patterns in subfalcine and uncal herniation may be anticipated from the fixed anatomical relationships between dural boundaries, adjacent brain structures and nearby arterial segments. These relationships define where vascular compromise is most likely to occur as a mass effect progresses.

### Falx–Cingulate–ACA Relationship

The falx cerebri forms a rigid midline dural boundary immediately adjacent to the cingulate gyrus and pericallosal ACA. Because the cingulate gyrus lies lateral to the falx and the ACA courses within the pericallosal cistern just inferior and lateral to the falcine edge, medial displacement can compress the vessel against this fixed barrier. The close spatial relationship between the falx, cingulate gyrus and ACA thus provides the anatomical basis for staged ACA compromise in subfalcine herniation (5–9), as shown in Figure 1, where the anterior falcine gap permits cingulate displacement while posterior falcine contact limits it.

### Tentorium–Uncus–PCA Relationship

The tentorium cerebelli forms a rigid dural boundary around the tentorial notch, creating a corridor through which the medial temporal lobe, oculomotor nerve and PCA are adjacent (10–13).

The uncus lies just above the tentorial edge, while the PCA, particularly the P2 segment, courses through the ambient cistern between the uncus and tentorium (14–17). During uncal herniation, medial temporal displacement may compress the PCA against the tentorial edge and lead to staged PCA ischaemia.

These anatomical relationships become clinically meaningful when expressed through morphometric parameters, such as a midline shift (MLS), interhemispheric corridor and incisural width. Variations in these parameters modify the displacement threshold at which arterial compression may occur.

### Anatomical Variability

Anatomical variations help explain why similar degrees of radiological displacement do not always produce the same infarction pattern. The most relevant modifiers in subfalcine and uncal herniation are the falx morphology and interhemispheric spacing, tentorial notch dimensions and arterial variants.

### Falx Types and Interhemispheric Spacing

Falx morphology may be grouped into three practical patterns based on the distance between the inferior falcine margin and the corpus callosum (18–20). A wider falx–callosal interval provides a larger interhemispheric buffer and may delay ACA compression, whereas a narrow corridor predisposes patients to earlier ACA compromise. Table 1 provides a summary of these practical morphometric categories.

### Tentorial Notch Types and Middle Incisural Space

Although morphometric studies describe multiple tentorial notch subtypes, they may be simplified for clinical use into small, typical and large configurations based on the notch width and available cisternal space (21, 22). A smaller notch creates a tighter anatomical bottleneck and may create a predisposition to earlier PCA compression, whereas a larger notch provides a wider corridor and may delay vascular compromise. Summaries of these practical categories are presented in Table 2 and Figure 2.

### ACA and PCA Variants

Although the general sequence of arterial compression is anatomically constrained, vascular variants may modify the laterality and extent of infarction. In subfalcine herniation, ACA variants, such as A1 hypoplasia or aplasia and an azygos ACA, may convert unilateral compression into bilateral parasagittal infarction. In uncal herniation, PCA variants, including foetal-type PCA and anterior choroidal anastomotic patterns, may alter the final occipitotemporal or deep ischaemic distribution (23).

## Haemodynamic Framework for Herniation-Related Infarction

ICP influences cerebral perfusion by lowering the transmural pressure and cerebral perfusion pressure (CPP = MAP – ICP). As ICP rises, arterial narrowing, increased vascular resistance and reduced cerebral blood flow follow; once autoregulation fails, perfusion becomes pressure-passive (24–30). The relationship between ICP, CPP and cerebral blood flow, which is summarised in Table 3, shows a progressive reduction in perfusion from early compromise to complete arterial collapse.

In herniation, global perfusion failure alone does not explain the infarct pattern. Directional tissue shift adds a second mechanism: the focal compression of arteries against rigid dural boundaries. As shown in Table 3, similar CPP thresholds may be associated with different clinical outcomes depending on the presence and direction of the tissue displacement. In practical terms, rising ICP reduces overall perfusion, whereas the direction and extent of displacement determine which vascular territory is most vulnerable. This combined haemodynamic–mechanical model explains why subfalcine displacement preferentially threatens the ACA and uncal displacement preferentially threatens the PCA.

## Stage-Dependent Arterial Compression in Brain Herniation

The MLS ranges were adapted from previously reported imaging-based categorisations and reorganised into a four-stage framework to correlate the displacement with likely vascular compromise (31). Table 4 provides a simplified summary of this anatomy-based framework.

### Stage-Dependent Compression of the ACA in Subfalcine Herniation

Subfalcine herniation produces a stage-dependent pattern of ACA compression driven by progressive medial displacement of the cingulate gyrus beneath the falx cerebri. This reflects the fixed anatomical relationship between the pericallosal ACA segments and the falx, whereby an increasing MLS results in sequential vascular compromise. The infarction patterns are categorised from Type 0 to Type E, which represent the progressive involvement of ACA territories with the increasing severity of arterial compromise. Type 0 indicates no infarction, while Types A–E reflect a stepwise extension from A2 hypoperfusion to multisegmental or bilateral ACA infarction. These relationships are summarised in Figure 3 and Table 5.

#### Stage 1 (MLS ≤ 5 mm): Early A2 Involvement

In the earliest stage, displacement is primarily limited to the anterior cingulate cortex. The A2 segment may undergo mechanical stretching and early compression, resulting in reduced perfusion. This corresponds to Type 0 (no infarction) or early Type A (A2 hypoperfusion), depending on individual anatomical constraints.

#### Stage 2 (MLS 6 mm to 10 mm): A2 Compression with Emerging A3 Involvement

With an increasing MLS, further medial displacement brings the cingulate gyrus and callosal genu into contact with the falx. A2 compression becomes more pronounced, and the A3 segment starts to become involved. This produces a transitional pattern of ischaemia, which corresponds to Type A–B infarction (A2 ± A3 involvement) (31).

#### Stage 3 (MLS 11 mm to 15 mm): Combined A2 + A3 Compromise

At this stage, further medial displacement results in sustained compression of the ACA against the falx, with involvement of both the A2 and A3 segments and possible early extension to the distal branches. This corresponds to Type C infarction (combined A2–A3 involvement), reflecting established medial frontal and callosal ischaemia. Clinically, more pronounced motor and behavioural deficits may occur due to the involvement of medial motor and limbic structures (32).

#### Stage 4 (MLS 16 mm to 20 mm): Multisegmental ACA Involvement (A2–A5 ± A1)

In advanced subfalcine herniation, progressive displacement may lead to extensive distal ACA compression (A4–A5) in addition to A2 and A3 involvement, with possible proximal extension to A1 in severe or anatomically constrained cases. This corresponds to Type D–E infarction and represents multisegmental or bilateral ACA territory involvement. In the presence of anatomical variants, such as an azygos ACA or A1 hypoplasia, bilateral infarction and severe clinical syndromes, including akinetic mutism, may occur (33).

Overall, ACA compression in subfalcine herniation may follow a distal-to-proximal sequence, typically beginning at A2 and progressing to more distal and, in severe cases, proximal segments. The displacement threshold for each stage is likely modified by the falx morphology and interhemispheric spacing.

### Stage-Dependent Compression of the PCA in Uncal Herniation

Uncal herniation, which is shaped by fixed anatomical relationships and the available middle incisural space, may produce a staged sequence of neurovascular compression within the tentorial incisura (34–37). The uncus, oculomotor nerve, PCA and cerebral peduncle are closely stacked within a narrow corridor; with progressive medial temporal displacement, compression is expected to involve these structures in sequence. Table 6 provides a summary of the overall staging, and Figure 4 illustrates the relevant anatomy.

Compared with the ACA, PCA involvement is less amenable to multiple large-stage subdivisions because compression is centred on the shorter P2 segment. Nevertheless, a proximal-to-distal pattern may still be anticipated: Early compromise of the P2 perforators and posterior choroidal branches may precede more distal P3–P4 cortical branch involvement, which would explain occipital infarction and homonymous visual field loss better (38). This proposed branch-based progression is summarised in Table 7.

This microstaging model is intended as an anatomical and pathophysiological framework rather than a definitive clinical staging system. In acute practice, branch-specific deficits may be obscured by reduced consciousness and evolving brainstem dysfunction. The main value of the framework is to help interpret imaging and anticipate which territories may be at risk during progressive uncal displacement.

## Case-Based Anatomical Demonstration

An 18-year-old man sustained severe traumatic brain injury following a motor vehicle accident and presented in a comatose state with a Glasgow Coma Scale score of 5 and bilaterally fixed, dilated pupils. Non-contrast CT demonstrated a left fronto-temporo-parietal acute subdural haematoma measuring approximately 0.5 cm in maximal thickness, with a rightward MLS of 8 mm and effacement of the basal cisterns (Figure 5).

The measured MLS of 8 mm corresponded to Stage 2 subfalcine herniation in the proposed framework, indicating displacement beyond the anterior cingulate cortex into the mid-cingulate and callosal regions. At this stage, the A2 and A3 segments of the ACA are expected to be the most vulnerable to compression at the falcine edge. In keeping with this pattern, CT demonstrated bilateral parasagittal hypodensities in the ACA territory, consistent with combined A2–A3 infarction involving the pericallosal trunk and its cortical distribution (Figure 6). The bilateral distribution suggested substantial mechanical compromise of the pericallosal circulation despite a measured MLS within the Stage 2 range.

The concurrent effacement of the basal cisterns indicated advanced uncal herniation, corresponding to at least Stage 3 neurovascular involvement. In this setting, medial temporal displacement within the tentorial incisura would be expected to compress the PCA progressively along its course, beginning at the P2 segment and extending distally with increasing displacement. Imaging demonstrated occipital hypodensities consistent with PCA territory infarction involving the parieto-occipital and calcarine distributions, which indicated distal cortical branch involvement at the P3–P4 level (Figure 7). This pattern is more consistent with progressive mechanical compression along the PCA than with isolated proximal branch compromise.

CT angiography demonstrated preserved opacification of the major intracranial arteries without evidence of large-vessel occlusion (Figure 8). This supported secondary vascular compromise from herniation-related mechanical distortion rather than primary thromboembolic occlusion. Taken together, the case demonstrates simultaneous stage-dependent arterial injury in two compartments: Stage 2 subfalcine herniation with A2–A3 ACA infarction and advanced uncal herniation with distal PCA cortical infarction. The extent of the infarction relative to the measured MLS further supports the influence of individual anatomical variations in the interhemispheric and incisural spaces on the severity and pattern of vascular compromise.

## Conclusion

Brain herniation may produce anatomically patterned arterial compromise, with subfalcine displacement primarily threatening the ACA and uncal displacement primarily threatening the PCA. The threshold and extent of vascular injury are modified by individual variations in interhemispheric and incisural anatomy, which makes morphometric parameters clinically relevant to infarct anticipation. The proposed anatomy-based framework may facilitate radiological interpretation, support the early recognition of herniation-related ischaemia and provide a practical lens for clinical decision making.

## Figures and Tables

**Figure 1:**
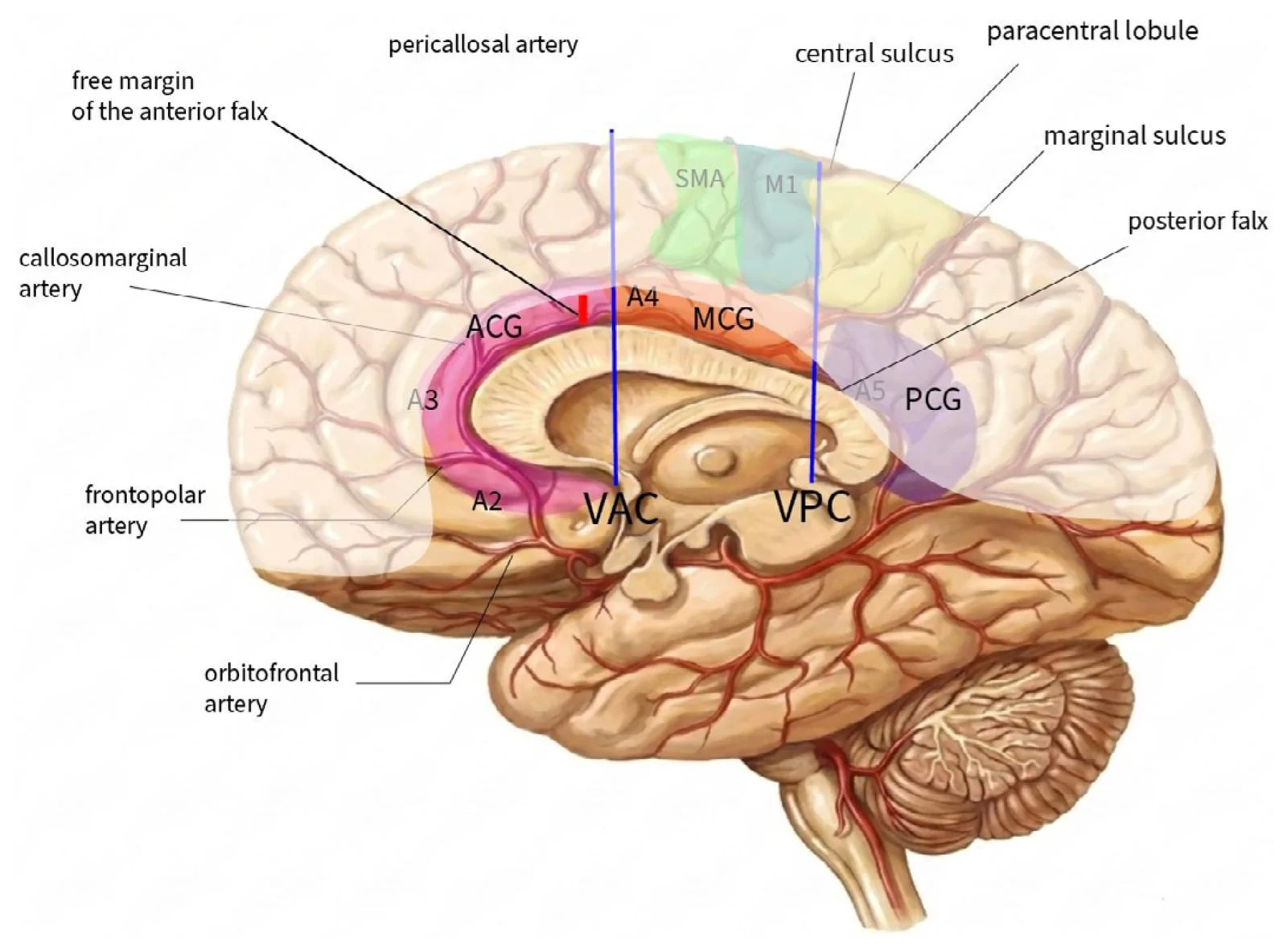
Relationship of the falx cerebri to the corpus callosum and cingulate gyrus. Anteriorly, a gap between the inferior free margin of the falx and the corpus callosum permits medial displacement of the cingulate gyrus in subfalcine herniation. Posteriorly, the falx approaches the splenium, limiting posterior displacement. The cingulate gyrus is divided into anterior, middle, and posterior segments by the vertical anterior commissure and vertical posterior commissure lines.

**Figure 2:**
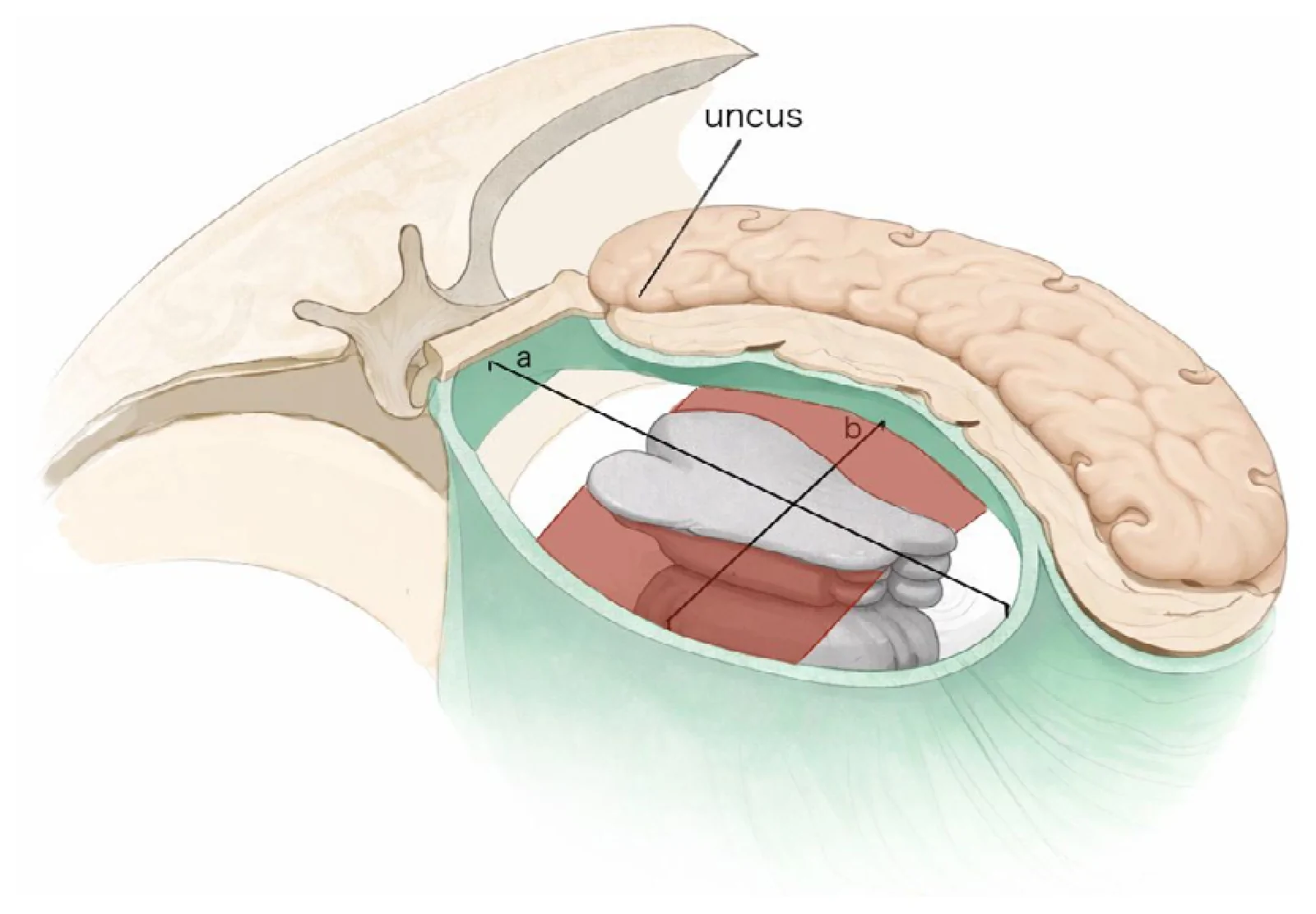
Tentorial incisural space and its relevance to uncal herniation, schematic illustration of the tentorial incisura. The red-shaded area represents the middle incisural space surrounding the midbrain. The uncus forms the anterior supratentorial boundary. (a) Maximum notch length, representing the anteroposterior dimension. (b) Maximum notch width, representing the transverse distance between free tentorial edges. Variations in these dimensions influence the available space for displacement and the threshold for neurovascular compression during uncal herniation.

**Figure 3:**
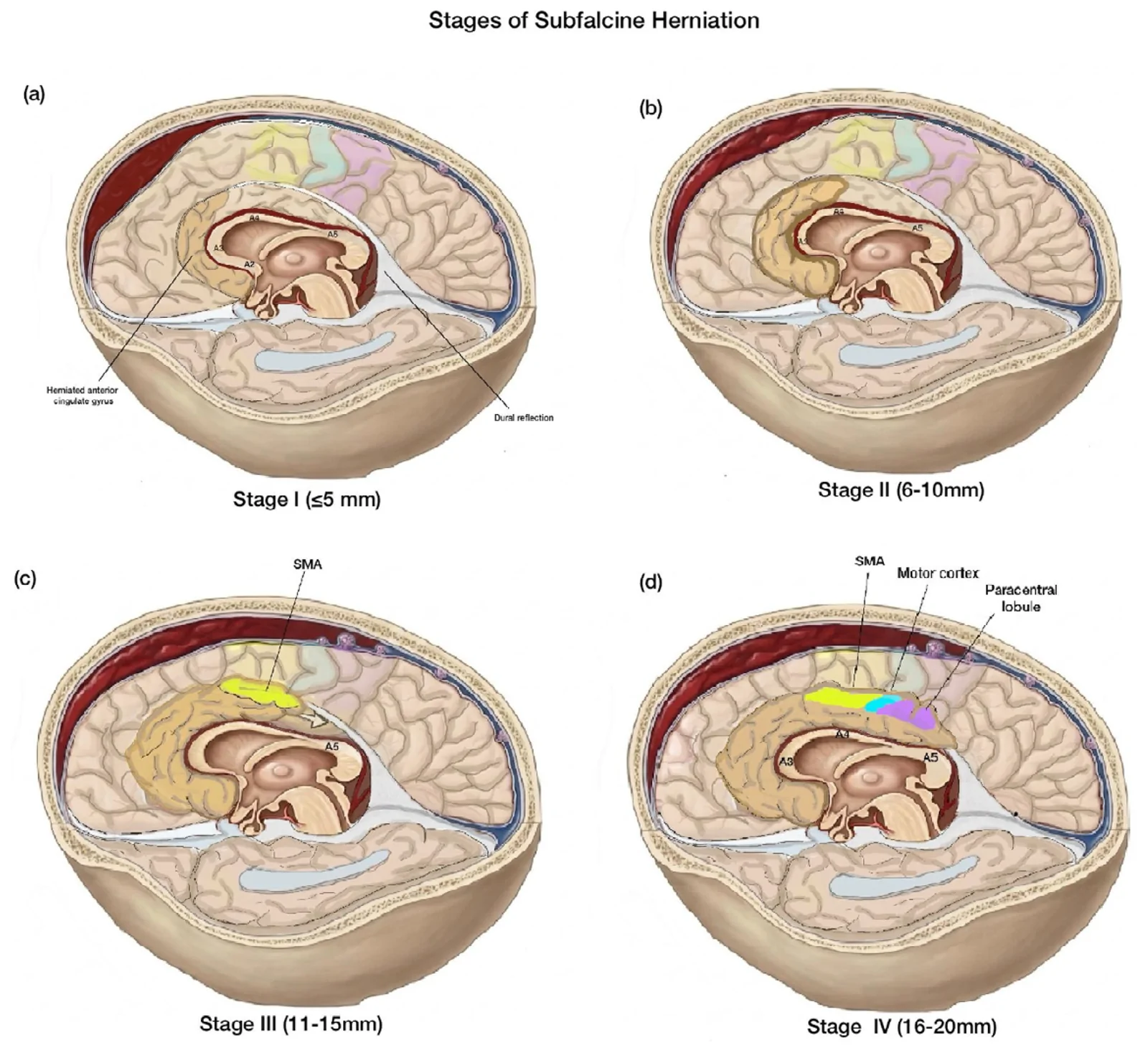
Progressive stages of subfalcine herniation and corresponding anatomical involvement. Schematic illustration of stage-dependent medial displacement. (a) Stage I: anterior cingulate cortex displaced beneath the falx. (b) Stage II: involvement of the mid-cingulate cortex, genu of the corpus callosum, and anterior medial frontal gyrus. (c) Stage III: extension to the supplementary motor area. (d) Stage IV: involvement of the corpus callosum body and isthmus, supplementary motor area, paracentral lobule, and posterior cingulate gyrus. This progression reflects increasing compression of pericallosal ACA segments with advancing MLS.

**Figure 4:**
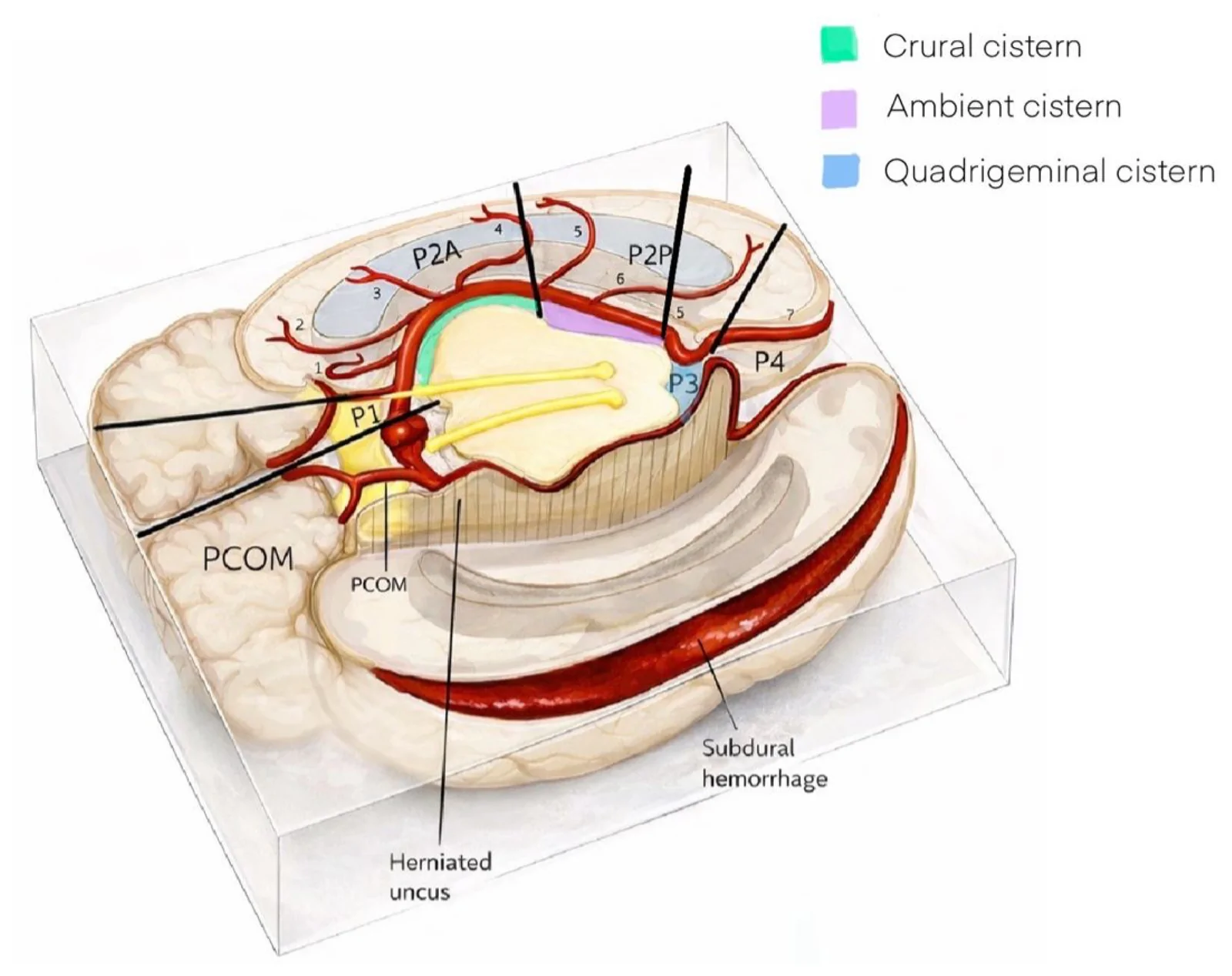
Relationship of PCA segments to the incisural cisterns and their principal branches. Schematic illustration of the PCA course within the crural and ambient cisterns in relation to the tentorial edge and medial temporal lobe. Numbered branches include: (1) uncal (hippocampal) artery; (2) anterior temporal artery; (3) middle temporal artery; (4) medial posterior choroidal artery; (5) lateral posterior choroidal artery; (6) posterior temporal artery; and (7) calcarine artery. This anatomical arrangement underlies the proximal-to-distal sequence of vascular compromise during uncal herniation.

**Figure 5:**
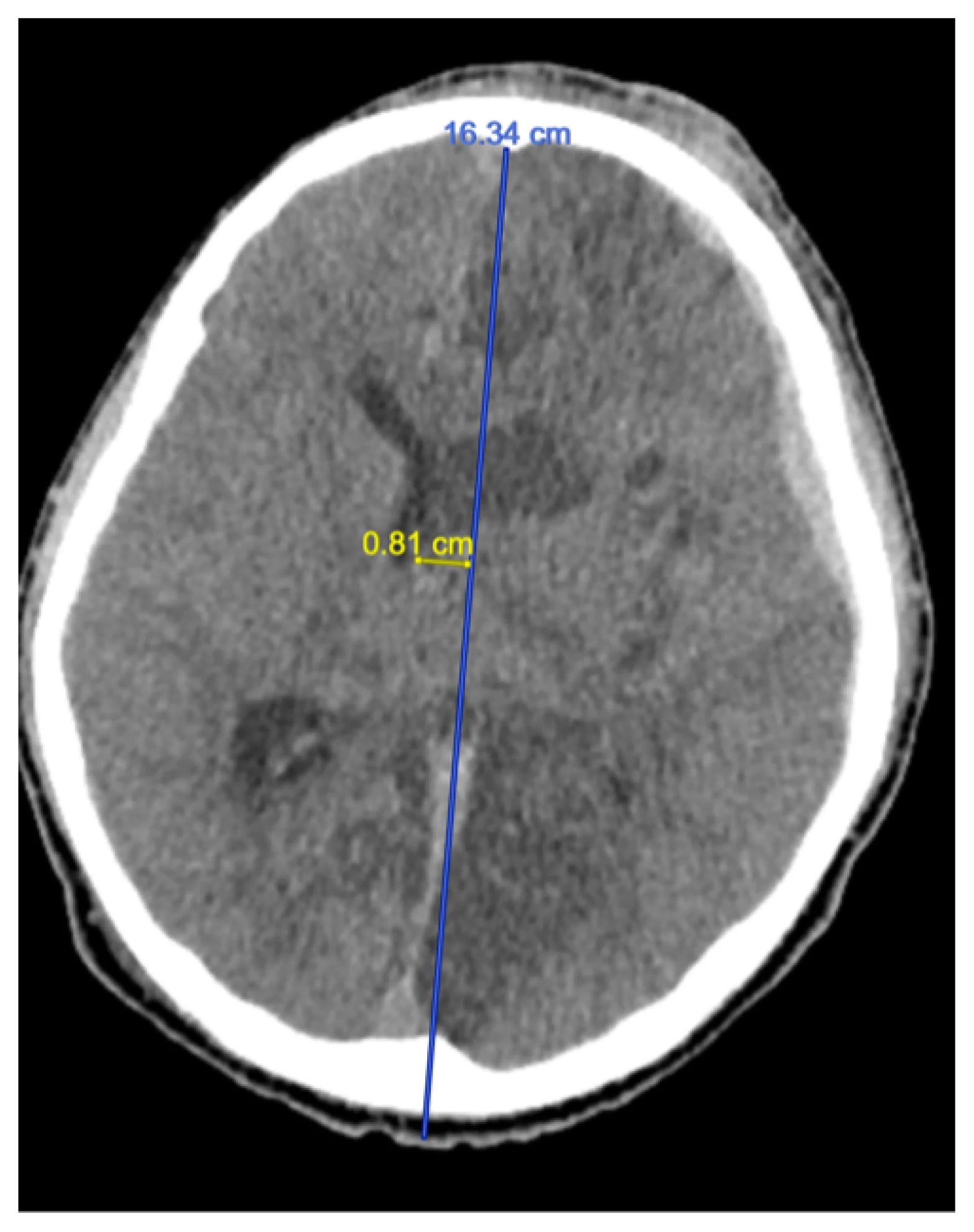
Non-contrast CT demonstrating a left acute subdural hematoma with approximately 8 mm MLS, with effacement of the basal cisterns and early ischaemic changes. The degree of MLS is consistent with Stage 2 subfalcine herniation within the proposed framework.

**Figure 6:**
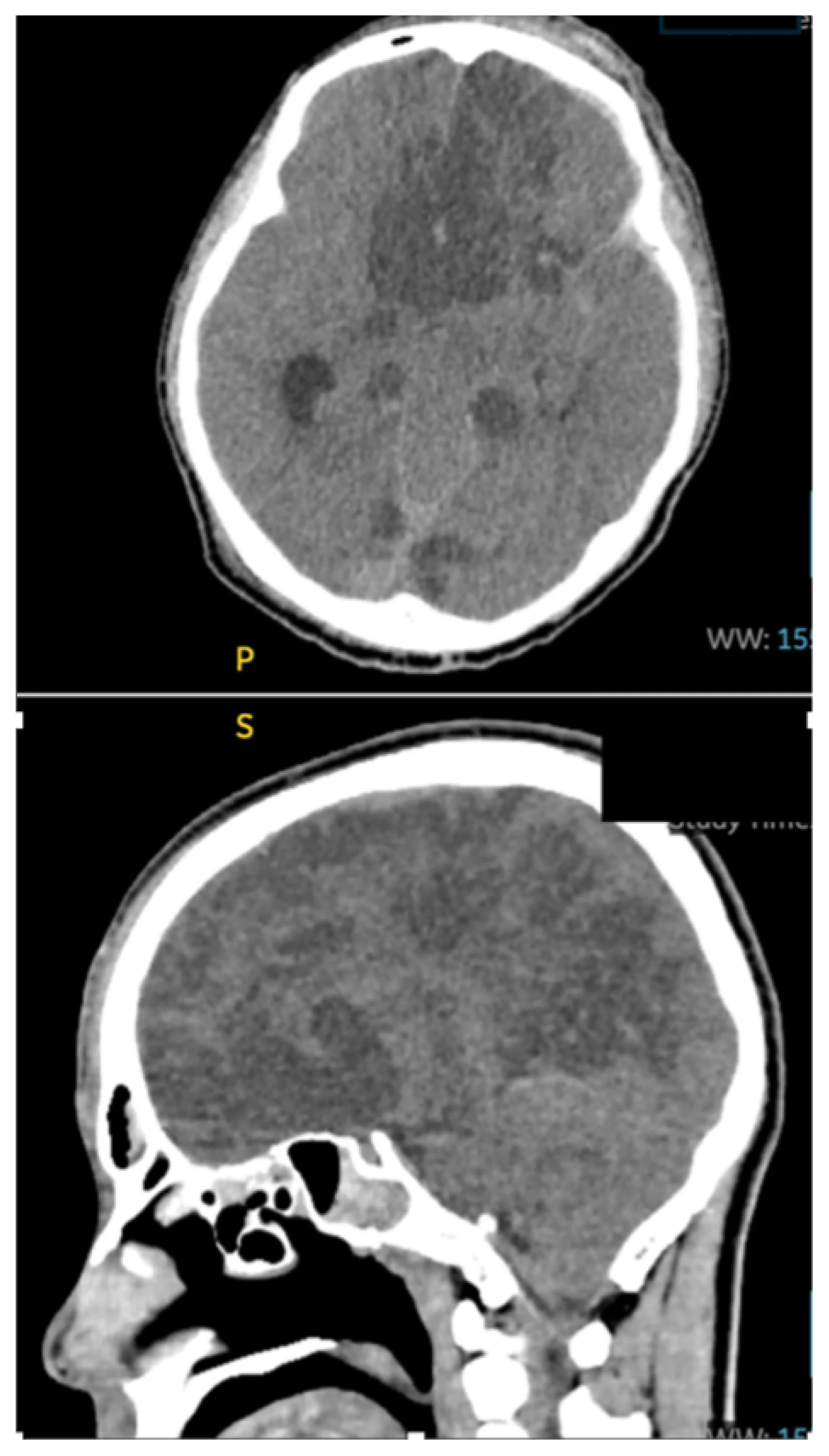
CT demonstrating bilateral parasagittal hypodensities in the ACA territory. This pattern is compatible with A2–A3 segment involvement within the proposed framework, corresponding to medial frontal and callosal infarction (Type C pattern).

**Figure 7:**
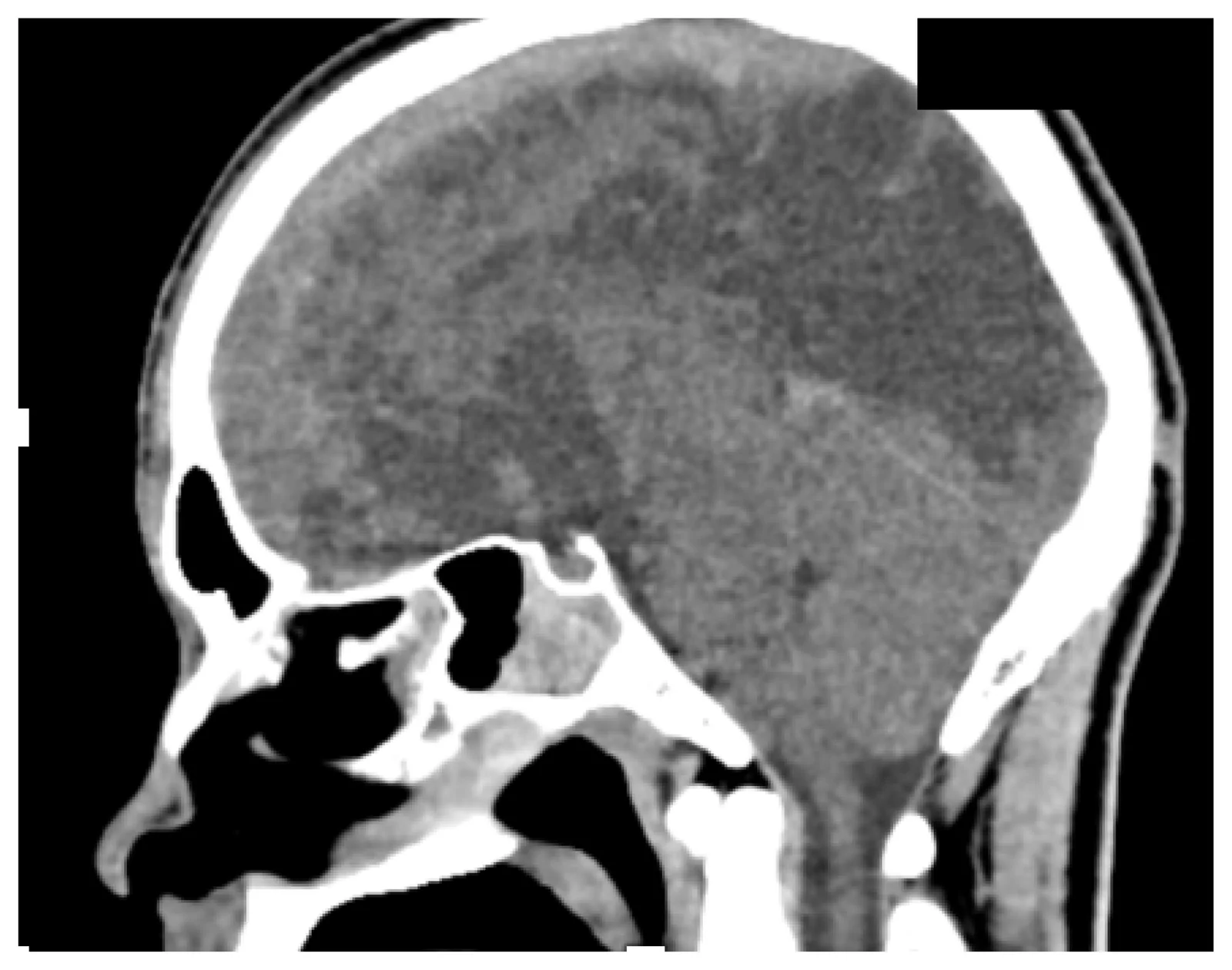
CT demonstrating occipital lobe hypodensities consistent with PCA territory infarction. This pattern is compatible with advanced uncal herniation within the proposed framework, involving distal P3–P4 cortical branches.

**Figure 8:**
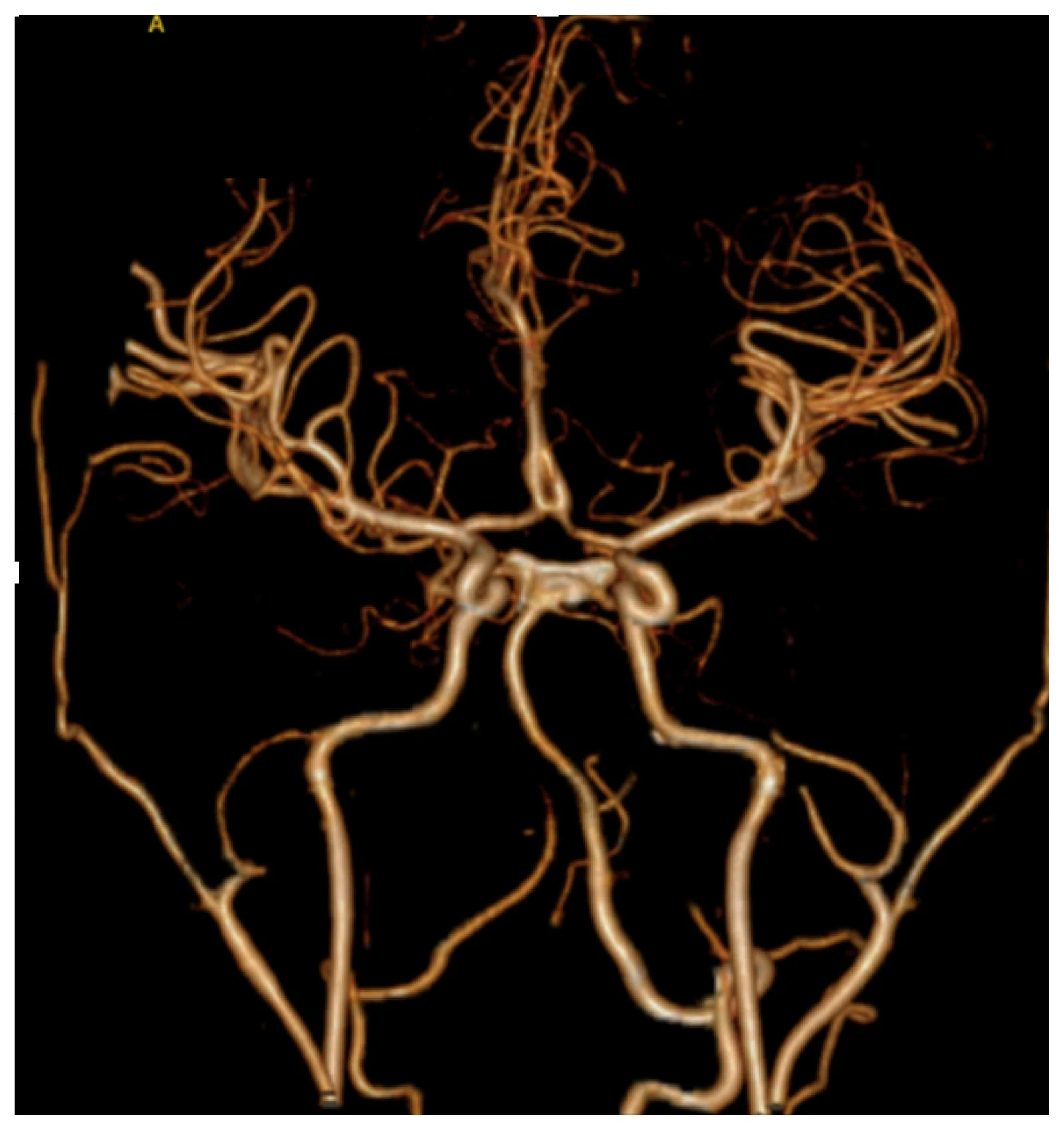
CT angiography demonstrating preserved opacification of major intracranial arteries without evidence of large-vessel occlusion. These findings support secondary vascular compromise due to herniation-related mechanical distortion rather than primary thromboembolic occlusion.

**Table 1:** Falx cerebri types based on falx-corpus callosum distance.

Type	Morphology	Corridor size	Distance (Falx–CC), mm	Frequency, %
Type I	Falx high, wide anterior gap	Wide	> 10	47
Type II	Falx follows CC	Moderate	3 to 10	21
Type III	Falx nearly touching CC	Narrow	< 3	32

**Table 2:** Tentorial notch type and middle incisural space morphometry.

Type	Morphology	Corridor size	MNW, mm	Estimated middle incisural space, mm	Frequency, %
Small notch	Narrow, steep tentorial edges; constricted incisura	Restricted corridor	25 to 28	8 to 10	~ 10 to 20
Typical notch	Balanced, oval-shaped notch with moderate dimensions	Moderate corridor	28 to 30	10 to 15	~ 40 to 60
Large notch	Wide, shallow tentorial opening; expanded incisura	Wide corridor	30 to 34	15 to 20	~ 20 to 35

MNW = maximum notch width

**Table 3:** Relationship between intracranial pressure (ICP), cerebral perfusion pressure (CPP), and cerebral blood flow (CBF) across stages of cerebral ischaemia.

Stage	ICP, mmHg	CPP, mmHg	CBF, mL/100 g/min
Normal	5 to 15	70 to 90	~ 50 to 60
Early compromise	20 to 25	50 to 70	~ 20 to 30
Ischaemia	30 to 40	< 50	< 20
Infarction	40 to 60	< 40	≤ 12
Arterial collapse	~ 60 to 80	~ 0	0

ICP = intracranial pressure; CPP = cerebral perfusion pressure; CBF = cerebral blood flow

**Table 4:** Four stages of MLS.

Stage	MLS, mm
1	≤ 5
2	6 to 10
3	11 to 15
4	16 to 20

**Table 5:** Stage-dependent ACA involvement and infarction patterns in subfalcine herniation according to falx type.

Falx type	Stage	ACA segment involved	Infarct pattern
Type I	1	None	Type 0
	2	A2 (stretch)	Type A
	3	A2 ± A3	Type A/B
	4	A2 + A3	Type C
Type II	1	A2	Type A
	2	A2 + A3	Type A/B
	3	A2 + A3 ± A4	Type C
	4	A2 to A5	Type D
Type III	1	A2 (early)	Type A
	2	A2 to A4	Type C
	3	A2 to A5 ± A1	Type D
	4	A1 to A5	Type E

ACA = anterior cerebral artery; Infarction patterns (Type 0–E) reflect progressive ACA compromise from A2 to distal and, in severe cases, proximal segments.

**Table 6:** Stage-dependent neurovascular compression in uncal herniation according to middle incisural space type.

Incisural space	Stage	Structure compressed	Key clinical feature
Small	1	CN III (parasympathetic)	Ipsilateral pupillary dilation
	2	CN III (complete)	Ptosis, ophthalmoplegia
	3	PCA (P2)	Homonymous hemianopia
	4	Cerebral peduncle	Hemiparesis
Typical	1 to 4	Same sequence (delayed threshold)	Same pattern
Large	1 to 4	Same sequence (further delayed)	Same pattern

The sequence of compression is preserved across anatomical variants, while the threshold for each stage varies with incisural space dimensions.

**Table 7:** Stage-dependent sequence of PCA branch involvement during uncal herniation.

Stage	PCA segment	Branch group	Territory	Clinical pattern
1	Proximal P2A	Perforators + choroidal	Midbrain, thalamus	↓ consciousness, memory
2	Distal P2A	Temporal branches	Temporal lobe	Subtle visual deficits
3	P2P	Posterior temporal + choroidal	Posterior temporal	Visual processing deficit
4	P3–P4	Cortical branches	Occipital cortex	Homonymous hemianopia

PCA = posterior cerebral artery; Progressive compression follows a proximal-to-distal pattern from P2 perforators to distal cortical branches.
